# Study of Repetitive Movements Induced Oscillatory Activities in Healthy Subjects and Chronic Stroke Patients

**DOI:** 10.1038/srep39046

**Published:** 2016-12-15

**Authors:** Chuan-Chih Hsu, Wai-Keung Lee, Kuo-Kai Shyu, Hsiao-Huang Chang, Ting-Kuang Yeh, Hao-Teng Hsu, Chun-Yen Chang, Gong-Yau Lan, Po-Lei Lee

**Affiliations:** 1 Division of Cardiovascular Surgery, Taipei Medical University Hospital, Taipei, Taiwan; 2 Department of Rehabilitation, Tao Yuan General Hospital, Taoyuan, Taiwan; 3 Department of Electrical Engineering, National Central University, Taoyuan City 32001, Taiwan; 4 Division of Cardiovascular Surgery, Taipei Veterans General Hospital, Taipei, Taiwan; 5 Science Education Center, National Taiwan Normal University, Taipei, Taiwan; 6 Section of General Diagnostic Radiology, Taipei Medical University Hospital, Taipei, Taiwan

## Abstract

Repetitive movements at a constant rate require the integration of internal time counting and motor neural networks. Previous studies have proved that humans can follow short durations automatically (automatic timing) but require more cognitive efforts to track or estimate long durations. In this study, we studied sensorimotor oscillatory activities in healthy subjects and chronic stroke patients when subjects were performing repetitive finger movements. We found the movement-modulated changes in alpha and beta oscillatory activities were decreased with the increase of movement rates in finger lifting of healthy subjects and the non-paretic hands in stroke patients, whereas no difference was found in the paretic-hand movements at different movement rates in stroke patients. The significant difference in oscillatory activities between movements of non-paretic hands and paretic hands could imply the requirement of higher cognitive efforts to perform fast repetitive movements in paretic hands. The sensorimotor oscillatory response in fast repetitive movements could be a possible indicator to probe the recovery of motor function in stroke patients.

Timing in the brain has its important role in many aspects, such as speech perception, speech production, reading, attention, memory, cognitive processing, decision-making, and motor coordination^1^. Especially, internal time counting is crucial for motor control in our daily life activities. The processing of time estimation for movements has been studied in many literatures^2^. Morillon *et al*. postulated the time estimation in human motor system as a dual system, which can track a short duration automatically (automatic timing) but requires more cognitive demands to track a long duration by a so-called default mode network (DMN)^3^. Poppel E. studied the capability of time estimation in a stimulus reproduction task from 0.5 s to 7 s, and found movements become temporally irregular for inter-movement interval (IMI) above 3 s which indicated precisely control of movements with IMIs longer than 3 s is not possible^4^. Though these literatures have shown great difference between movements in short and long durations in healthy subjects, nevertheless, the study of brain responses induced by rapid movements in patients with motor neurological disorder was seldom reported.

Several imaging modalities have been developed to quantify motor response in human brain, including EEG, MEG, fMRI, TMS, etc.^5^,^6^. The EEG, which is the tool used most widely, has the advantages of low-cost, easy preparation, and its superiority of high temporal resolution to measure fast changes of neural oscillatory activities. Neural oscillatory activities in human brain can be either phase-locked or non-phase-locked reactive to external or internal stimuli. These oscillatory activities usually exist in specific frequency bands and spatial locations. Event-related non-phase-locked neural activities represent power changes, either enhanced or suppressed relative to baseline activities. The power changes in event-related activities can be caused by the decrease or increase in synchrony of the underlying activated neuronal populations. Pfurtscheller *et al*.^7^ studied the Mu-rhythm changes in discrete voluntary finger movements, and found oscillatory activities were suppressed, started about 1.5 s preceding movement onsets, followed by post-movement power rebound, occurred around 0.7 s~1 s after movement offsets^7^. The power suppression was referred to as event-related desynchronization (ERD), reflecting the motor planning and preparation of initialization a movement, whereas the post-movement power rebound was referred to as event-related synchronization (ERS), indicating the motor inhibition or idling of motor neural network. Other EEG techniques, such as temporal-spectral evolution (TSE)^8^, amplitude modulation (AM)^9^, autoregression model method (AR)^10^, etc., were also developed to quantify task-specific brain oscillatory activity. These signal processing tools enable researchers to quantify the neural activities under different experimental manipulations and provide evidences for diagnosing clinical neurological diseases^11^,^12^,^13^.

The difference of brain oscillatory activities between healthy and stroke patients has been investigated in some studies. Rossiter *et al*. studied the movement-related beta desynchronization (MRBD) in healthy and middle cerebral artery (MCA) stroke patients^14^. They found reduced MRBD when patients were performing visually-cued grip task with their affected hand, compared to the MRBD obtained from healthy subjects. Giaquinto *et al*. followed up the changes of resting EEG in different frequency bands over six months in MCA stroke patients^15^, and they observed the amplitudes of movement-related Mu – rhythm improved significantly in the first three months and reached stable states in six months after stroke. Tecchio *et al*. studied the rhythmic brain activity at resting states in mono-hemispheric MCA stoke patients^16^. They found both the values of spectral power in affected and unaffected hemispheres were increased over Rolandic areas. Stepien *et al*. studied alpha ERD in stroke patients with cortical and subcortical lesions in performing a visually-cued button press task^17^. They found suppressed ERD in affected hemisphere when moving paretic hand, while no suppression in alpha ERD was found in the affected hemisphere when moving non-paretic hand. These studies measured oscillatory activities of sensorimotor Mu rhythm in visual selection task or slow self-paced voluntary movement (IMI ≥ 7 s). Oscillatory activity induced by fast repetitive movement in stroke patient was not studied. Since fast simple movement has been reported to have strong coupled connections among motor-related cortices^18^, study of cortical oscillatory activity in rapid simple movements could be crucial for the understanding of motor function in stroke patients.

Fast repetitive movement with short IMI recruits several motor-related areas in human brain, including primary motor cortex (M1), premotor cortex, supplementary motor cortex, cingulate cortex, basal ganglia, and thalamus^19^. Studies in healthy subjects have shown clear difference between the oscillatory activities induced by slow and fast repetitive movements. Wu *et al*. recorded the post-movement beta rebound (PMBD) in healthy subjects and observed that the PMBD was suppressed with the decrease of IMI in repetitive finger-lifting movements^19^. Erbil and Ungan^19^ investigated EEG alpha and beta oscillatory activities in repetitive extension-flexion finger movements over rolandic regions. Sustained suppression in Mu rhythm was observed during continuous movements which indicated that continuous movements are conducted through neural processing distinct from discrete movements. Bortoletto and Cunnington measured the fMRI responses of repetitive movements, and compared the results with another two finger movements with highly cognitive demands, one was a complicated sequencing task and the other was a timing task^20^. They found neural activities in lateral prefrontal regions were participated differently in the three tasks, owing to the different levels of cognitive efforts involved in the three tasks. In this study, we aimed to study the oscillatory activities induced by simple repetitive movements in healthy subjects and chronic stroke patients. The difference of oscillatory activities between stroke patients and healthy subjects might be a potential feature to evaluate the recovery of motor function in stroke patients.

## Materials and Methods

Twenty hemiparesis chronic stroke patients, P1~P20 (aged 58.25 ± 6.81 years-old; fifteen males and five females), who suffered from first-ever monohemispheric ischemic stroke in middle cerebral artery (MCA) territories confirmed by brain CT or MRI, were recruited. Patients with other neurological disorders and those with language/cognitive deficits were excluded from our experiment. In addition, twenty healthy subjects, H1~H20 (aged 54.76 ± 7.54 years-old; fourteen males and six females), with no history of clinical cerebrovascular disease were also recruited for comparison purpose. Patients’ muscle strengths were confirmed by measuring hand grip strengths following the instruction of Wolf Motor Function Test (WMFT), which requested patients to grip a dynamometer with greatest possible grip strength. The grip strengths were measured by dynamometer using PowerLab system (MacLab, ADInstruments, Castle Hill, Australia). Three finger-lifting experiments with IMIs chosen as 3 s, 2 s and 1 s, referring to slow (0.33 Hz), moderate (0.5 Hz) and fast (1 Hz) movement rates, were designed. The demographic data of stroke patients are listed in Table 1.

In each experiment, subjects were requested to perform repetitive finger-lifting movements in two sessions, one for left hand movement and the other for right hand movement. Each session included a training phase and an execution phase. In the training phase, a sequence of repetitive beep sounds at a specified rate were presented to subjects for two minutes, and subjects were instructed to execute finger-lifting movements following the beep sounds of metronome. In the execution phase, subjects were requested to perform internal time counting to reproduce the finger-lifting movements at a designate rate for nine minutes, separated into three sub-sessions with one-minute rest between any two successive sub-sessions. For each movement, the duration between current movement onset and the previous one, denoted as inter-movement duration, was calculated. The research was carried out in compliance with Helsinki declaration. All subjects gave informed consent, and the study was approved by the Ethics Committee of Institutional Review Board (IRB), Taoyuan General Hospital, Taiwan.

EEG data were recorded by a 16-channel EEG system (band-pass, 0.05-100 Hz; 60 Hz band-stop; 1 kHz sampling rate; Vamp system, Brain Products Co., Munich, Germany). In this study, we focused on studying the oscillatory activities of sensorimotor Mu rhythms. Only two EEG channels, C3 and C4 were used, with an another reference electrode placed at right mastoid and a ground electrode placed at left mastoid. In addition, an electro-oculogram (EOG) was used to reject artifact-contaminated epochs, with two electrodes placed above (EOG+) left eye and outer right canthus (EOG-). Impedance was kept below 5 kΩ for all electrodes. The onset timing of finger movement (movement onset) was detected by an optical switch and recorded together with EEG data for the subsequent off-line analysis^21^. All the data were stored in hard disk and off-line analyzed using Matlab software (Mathworks Co., USA).

The recorded EEG signals were filtered (zero-phase, 6^th^-order IIR Butterworth filter) within alpha (8~14 Hz) and beta (15~30 Hz)^22^ bands to obtain alpha and beta oscillatory activities. The alpha and beta oscillatory activities were further rectified and smoothed with a 0.1 s moving-average window. The rectified signals were then segmented into epochs, from −1.5 s to 1.5 s, anchored to movement onsets. The inter-movement duration was examined for each epoch, and only those artifact-free (EOG < 300μv) epochs with inter-movement durations close to the designate IMI (deviation time < 0.5 s) were selected for the following averaging process. These selected epochs were averaged over every 60 epochs to obtain event-related oscillatory response. The amplitude response *Amp*_*resp*_ of the event-related oscillatory response was calculated by finding the difference between the maximum amplitude *Amp*_*max*_ within 0 s ~2.0 s and the minimum amplitude *Amp*_*min*_ within −1.5 s~0 s in accordance with the onset time of each finger movement.

**Figure 1:**
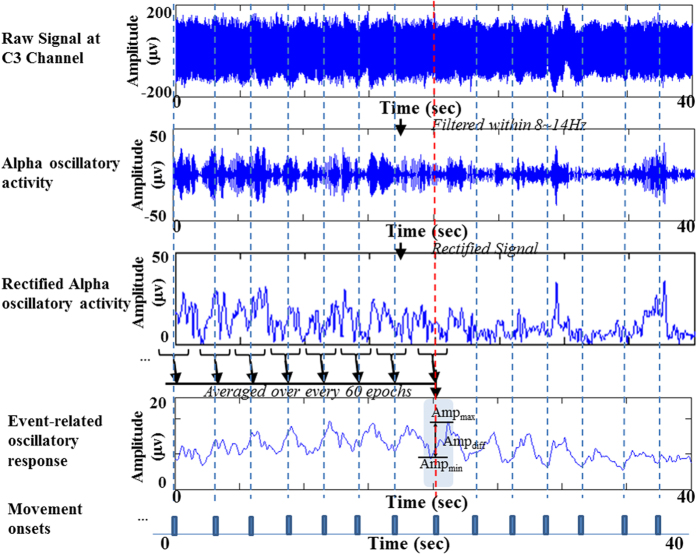
Demonstration of signal processing for quantifying event-related oscillatory response in subject H1.

Figure 1 shows one example of the signal processing for alpha *Amp*_*resp*_ at C3 channel in healthy subject H1. The beta *Amp*_*resp*_ can be obtained in the same procedure by filtering the raw EEG signal within beta band. In Fig. 1, the first and second panels show the raw EEG recording and the oscillatory activity filtered within alpha band, respectively. The third panel is the signal envelope of the rectified alpha oscillatory activity using Hilbert transform – based amplitude modulation (AM) method^9^. The dashed lines indicate the movement onsets recorded by optical switch. The fourth panel presents the event-related oscillatory response corresponding to a movement trigger (marked by red dashed line), obtained by averaging 60 segmented epochs from rectified oscillatory activity. The *Amp*_*resp*_ was calculated by finding the amplitude difference between the *Amp*_*max*_ in post-movement segment and the *Amp*_*min*_ in the segment preceding the movement onset, *i.e., Amp*_*resp*_ = *Amp*_*max*_*−Amp*_*min*_. Since the baseline of EEG rhythm was reported to have its functional meaning^23^, the amplitude response *Amp*_*resp*_ was further normalized to the mean of event-related oscillatory response, which can be represented as


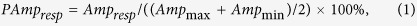


where *PAmp*_*resp*_ is the percentage of amplitude change relative to the mean of event- related oscillatory response.

It was reported that ipsilateral motor-related hyperactivity was observed in patients with cerebral occlusive disease^24^. Therefore, the laterality index (*LI*) was used to quantitatively describe the inter-hemispheric asymmetry^25^. The *LI* was calculated as the inter-hemispheric difference of *PAmp*_*resp*_ divided by the sum of *PAmp*_*resp*_ in both hemispheres. The calculation of *LI* is represented as follows,


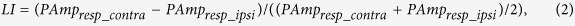


where *PAmp*_*resp_contra*_ and *PAmp*_*resp_ipsi*_ are the *PAmp*_*resp*_s obtained from the sensorimotor areas contralateral and ipsilateral to the movement hand, respectively.

The differences among the *PAmp*_*resp*_s obtained from the three movement rates were tested using one-way ANOVA. For one-way ANOVA tests reached significant levels (*p* < 0.05), two-tailed student’s *t*-test was chosen as post-hoc test to confirm the differences in different pairs of groups. Since each subject was requested to perform finger-lifting task at three different movement rates, thee pairs of group combinations, including slow-rate group *v.s*. medium-rate group, slow-rate group *v.s.* fast-rate group and medium-rate group *v.s.* fast-rate group, are tested in post-hoc tests. Taking multiple comparison problem (family-wise error) into consideration, the significance level (*p* < 0.05) was corrected as 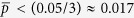 according to Bonferroni correction. Only the post-hoc tests which reached the corrected significant level 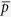 were marked in the following figures.

## Results

**Figure 2:**
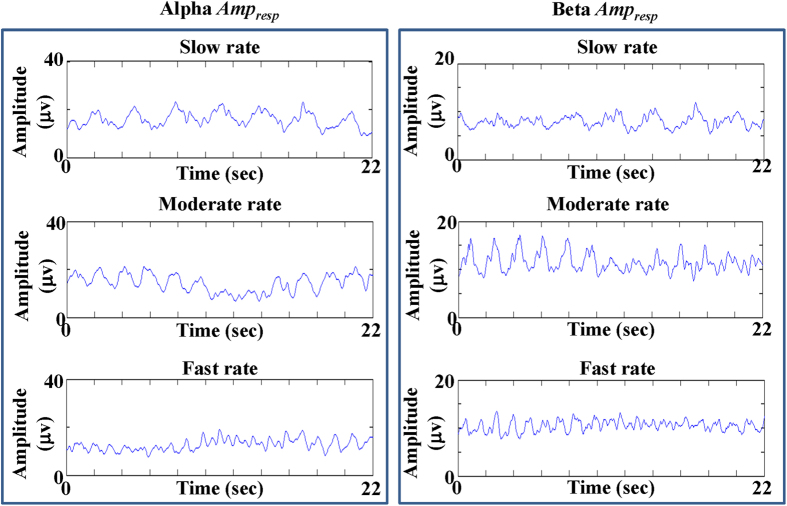
One example of the event-related oscillatory responses in alpha and beta bands at C3 channel when healthy subject N2 was performing repetitive finger movements at different rates.

Figure 2 presents one example of the event-related oscillatory responses in alpha and beta bands at C3 channel when healthy subject H2 was performing repetitive finger movements at different rates. In the left panels, the alpha *Amp*_*resp*_s were 24.51 ± 3.83 μv, 19.68 ± 5.3 μv and 11.39 ± 3.73 μv in slow-rate, moderate-rate and fast-rate movements, respectively. In the right panels, the beta *Amp*_*resp*_s were 9.46 ± 2.8 μv, 11.79 ± 3.63 μv, 6.43 ± 1.88 μv at slow-rate, moderate-rate and fast-rate movements, respectively. It can be observed that both the alpha and beta *Amp*_*resp*_ showed declined amplitude changes with the increase of movement rates.

**Figure 3:**
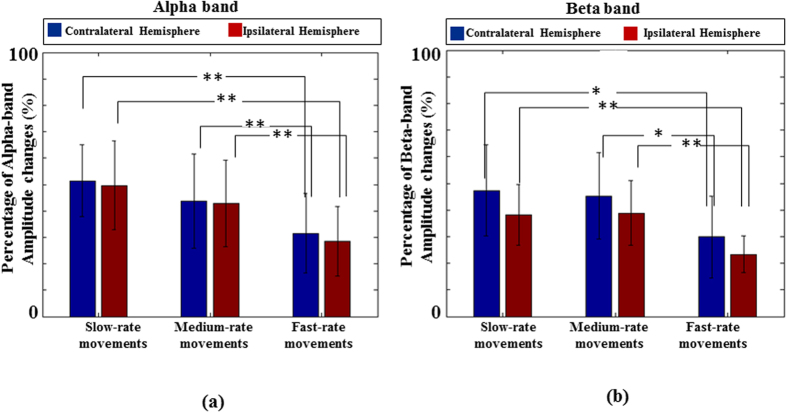
The alpha and beta *PAmp*_*resp*_s when the twenty healthy subjects were executing repetitive finger movements at different movement rates. (**a**) The alpha *PAmp*_*resp*_s from sensorimotor regions. (**b**) The beta *PAmp*_*resp*_s from sensorimotor regions. *p < 0.05; **p < 0.01.

Figure 3a,b show the alpha and beta *PAmp*_*resp*_s when the twenty healthy subjects were executing repetitive finger movements. Since this study aims to study the difference of alpha and beta *PAmp*_*resp*_s in healthy subjects with those obtained from non-paretic and paretic hand movements in stroke patients, the handness effect was not the main purpose of this study. Therefore, the data of right and left hand movements in healthy subjects were pooled together for the following statistical analyses. In Fig. 3a, the alpha *PAmp*_*resp*_s from sensorimotor regions were 51.41 ± 13.61%, 42.81 ± 17.87%, 31.58 ± 14.97% in the contralateral hemispheres for slow-rate, moderate-rate and fast-rate movements, respectively. In the ipsilateral hemispheres, the alpha *PAmp*_*resp*_s were 49.70 ± 16.73%, 42.84 ± 16.33%, 28.51 ± 13.16% in slow-rate, moderate-rate and fast-rate movements, respectively. Applying one-way ANOVA to examine the effect of movement rate on alpha *PAmp*_*resp*_s, significant differences were found among the three movement-rate groups in both the contralateral hemispheres (*p* < 0.01, F(2,57) = 8.25) and the ipsilateral hemispheres (*p* < 0.01, F(2,57) = 9.75). After further examining the alpha *PAmp*_*resp*_s of every two movement-rate groups, two-tailed student’s *t*-test showed significant differences in slow-rate group *v.s*. fast-rate group (*p* < 0.01 for both contralateral and ipsilateral hemispheres) and in medium-rate group *v.s*. fast-rate group (*p* < 0.01 for both contralateral and ipsilateral hemispheres).

In Fig. 3b, the beta *PAmp*_*resp*_s were 47.33 ± 17.05%, 45.25 ± 16.29%, 29.85 ± 15.41% in contralateral hemispheres for slow-rate, moderate-rate and fast-rate movements, respectively. The beta *PAmp*_*resp*_s in ipsilateral hemispheres were 38.20 ± 11.36%, 38.77 ± 12.12%, 23.31 ± 6.84% for slow-rate, moderate-rate and fast-rate movements, respectively. Significant differences were found among the three groups using one-way ANOVA in the contralateral hemispheres (*p* < 0.05, F(2,57) = 3.74) and the ipsilateral hemispheres (*p* < 0.01, F(2,57) = 6.34). Using two-tailed student’s *t*-test to examine every two groups, significant differences were found for the fast-rate movement group versus the other two movement-rate groups (*p* < 0.017 for contralateral hemispheres; *p* < 0.01 for ipsilateral hemispheres), while no significant difference was found between the groups of slow-rate movement and moderate-rate movement (*p* = 0.49 for contralateral side; *p* = 0.85 for ipsilateral side). Both the alpha and beta *PAmp*_*resp*_s demonstrated obvious amplitude suppressions in performing fast-rate movements.

**Figure 4:**
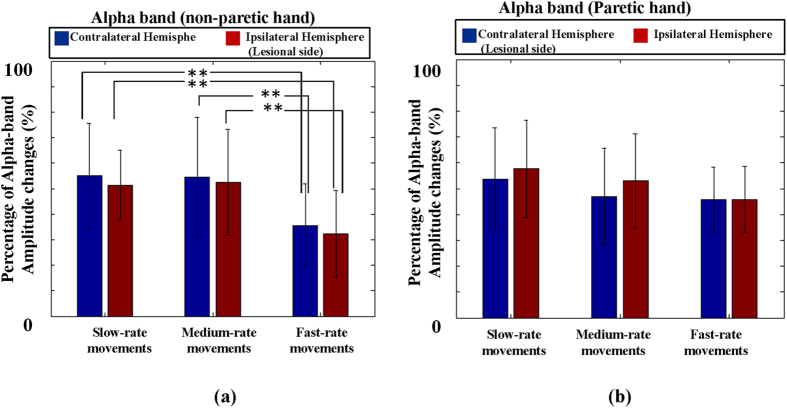
The alpha *PAmp*_*resp*_s induced from patients’ non-paretic hand and paretic hand movements in different movement rates. (**a**) The alpha *PAmp*_*resp*_s obtained from non-paretic hand movements. (**b**) The alpha *PAmp*_*resp*_s of paretic hand movements. **p < 0.01.

**Figure 5:**
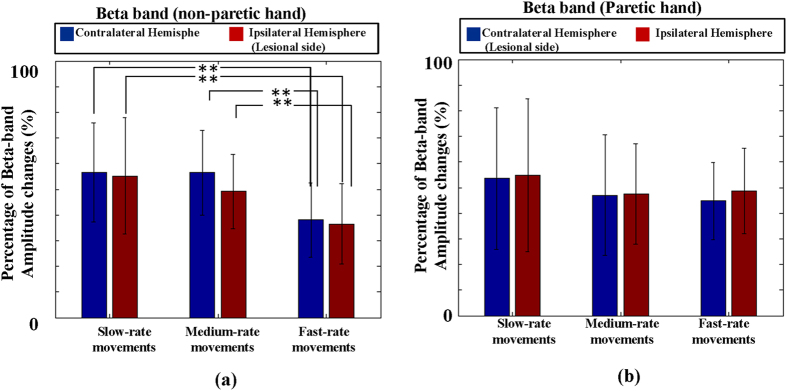
The beta *PAmp*_*resp*_s induced from patients’ non-paretic hand and paretic hand movements in different movement rates. (**a**) The beta *PAmp*_*resp*_s obtained from non-paretic hand movements. (**b**) The beta *PAmp*_*resp*_s of paretic hand movements. **p < 0.01.

Figures 4 and 5 show the alpha and beta *PAmp*_*resp*_s induced from patients’ non-paretic hand and paretic hand movements in different movement rates. In Fig. 4a, the alpha *PAmp*_*resp*_s obtained from non-paretic hand movements for slow-rate, moderate-rate and fast-rate movements were 55.06 ± 20.55%, 54.53 ± 29.10% and 35.63 ± 16.30% in contralateral hemispheres and were 51.43 ± 16.95%, 52.46 ± 20.73% and 32.32 ± 17.08% in ipsilateral (lesional) hemispheres, respectively. The results of one-way ANOVA showed statistical significance in both the contralateral (p < 0.05, F(2,57) = 4.38) and the ipsilateral (lesional) hemispheres (p < 0.05, F(2,57) = 3.3). Further examination using two-tailed student’s *t*-test showed significant differences in slow-rate group *v.s*. fast-rate group and in moderate-rate group *v.s*. fast-rate group (p < 0.01), while no difference was found between slow-rate group and moderate-rate group in both contralateral (*p* = 0.94) and ipsilateral (lesional) hemispheres (*p* = 0.86). Compared to the alpha *PAmp*_*resp*_s of paretic hand movements shown in Fig. 4b, the slow-rate, moderate-rate and fast-rate movements were 53.71 ± 19.80%, 46.94 ± 18.60%, 45.75 ± 12.64% in the contralateral (lesional) hemispheres and were 57.67 ± 18.84%, 52.95 ± 18.23%, 45.90 ± 15.78% in the ipsilateral hemispheres, respectively. No significant difference was found among the three movement-rate groups in contralateral (lesional) (*p* = 0.51, F(2, 57) = 0.67; one-way ANOVA) and ipsilateral hemispheres (*p* = 0.26, F(2, 57) = 1.35; one-way ANOVA).

In Fig. 5a, the beta *PAmp*_*resp*_s in patients’ non-paretic hand movements for slow-rate, moderate-rate and fast-rate movements were 56.95 ± 19.28%, 56.50 ± 16.55% and 38.05 ± 14.47% in contralateral hemispheres and were 55.18 ± 22.71%, 49.12 ± 14.58% and 36.47 ± 15.64% in ipsilateral (lesional) hemispheres. Using one-way ANOVA, the comparison among three groups had reached significant differences in both the contralateral hemisphere (*p* < 0.05, F(2,57) = 4.38) and the ipsilateral (lesional) (*p* < 0.05, F(2,57) = 3.64). Examining every pair of the three groups using two-tailed student’s *t*-test, significant levels were reached in slow-rate group *v.s.* fast-rate group (*p* < 0.01) and moderate-rate group *v.s*. fast-rate group (*p* < 0.01), but no significant level was found between slow-rate and moderate-rate groups in contralateral (*p* = 0.97) and in ipsilateral (lesional) hemispheres (*p* = 0.30). The beta *PAmp*_*resp*_s of the paretic hand movements in Fig. 5b were 53.55 ± 27.66%, 47.02 ± 23.56% and 44.79 ± 15.12% in contralateral (lesional) hemispheres and were 54.88 ± 29.82%, 47.48 ± 19.58% and 48.67 ± 16.76% in ipsilateral hemispheres for slow-rate, moderate-rate and fast-rate movements, respectively. No difference was found among the three groups in contralateral (lesional) (*p* = 0.65, F(2,57) = 0.44) and in ipsilateral hemispheres (*p* = 0.72, F(2,57) = 0.33).

**Figure 6:**
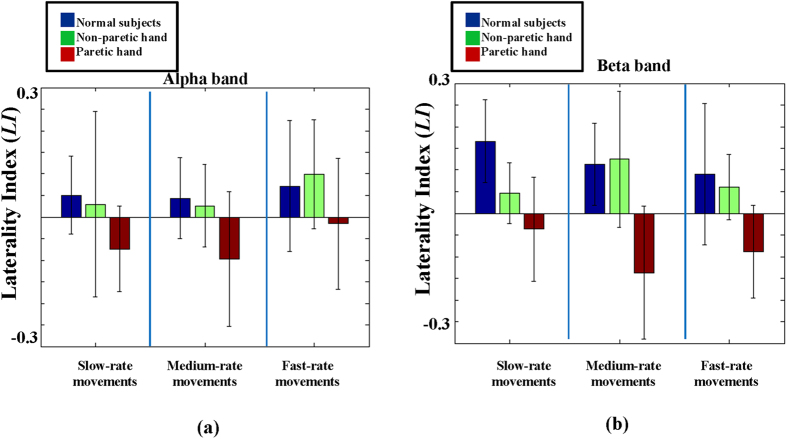
The comparison of *LI*s in hand movements of healthy subjects, and the non-paretic hand and paretic movements of stroke patients. (**a**) The *LI*s of alpha *PAmp*_*resp*_. (**b**) The *LI*s of beta *PAmp*_*resp*_.

Figure 6 shows the comparison of *LI*s in hand movements of healthy subjects, and non-paretic hand and paretic movements of stroke patients. In Fig. 6a, the *LI*s of alpha *PAmp*_*resp*_ were 0.05 ± 0.09, 0.03 ± 0.21 and -0.07 ± 0.09 in slow-rate movements, 0.04 ± 0.09, 0.03 ± 0.09 and -0.09 ± 0.15 in moderate-rate movements, and 0.07 ± 0.15, 0.10 ± 0.13, -0.01 ± 0.15 in fast-rate movements for healthy subject, and non-paretic and paretic hands in stroke patients, respectively. In Fig. 6b, the *LI*s of beta *PAmp*_*resp*_ were 0.16 ± 0.09, 0.04 ± 0.07 and -0.03 ± 0.12 in slow-rate movements, 0.11 ± 0.09, 0.12 ± 0.15 and −0.13 ± 0.15 in moderate-rate movements, and 0.09 ± 0.16, 0.06 ± 0.07, −0.09 ± 0.10 in fast-rate movements for healthy subjects, non-paretic and paretic hands in stroke patients, respectively.

In this study, only those artifact-free epochs with their inter-movement durations close to the designate IMI (deviation time < 0.5 s) were chosen for the *PAmp*_resp_ calculations. 7 shows the histograms of percentages of inter-movement durations executed by healthy and stroke subjects. In healthy subjects, the inter-movement durations were 2.82 ± 0.07 s, 1.96 ± 0.05 s and 1.10 ± 0.06 s for slow-rate, medium-rate and fast-rate movements, respectively. With the exclusion of movements with large deviation time (>0.5 s), the retention rates were 83.3%, 97.3% and 97.4% for slow-rate, medium-rate and fast-rate movements, respectively. In stroke patients, the inter-movement duration for non-paretic hand movements were 2.86 ± 0.09 s, 2.07 ± 0.07 s and 1.17 ± 0.06 s at slow-rate, medium-rate and fast-rate movements with retention rates of 53.1%, 83.4% and 88.6%, respectively. For paretic-hand movements, the inter-movement duration for non-paretic hand movements were 3.16 ± 0.10 s, 2.11 ± 0.12 s and 1.22 ± 0.12 s at slow-rate, medium-rate and fast-rate movements with retention rates of 38.2%, 45.1% and 76.9%, respectively.

## Discussion

This paper studied the brain oscillatory activities modulated by different rates of repetitive finger movements in healthy subjects and chronic stroke patients. In contrast to other literatures which chose acute stroke patients to participate in their studies^17^,^26^,^27^, we chose chronic stroke patients in our study owing to the consideration of the capability of stoke patients in producing a consistently repetitive movements. The execution of repetitive movements at a specific rate relies on the capability of internal time counting and the involvements of motor neural networks. Compared to other ERD/ERS with long IMIs which quantified induced oscillatory activities by finding the power (or amplitude) ratio between maximum suppression (or rebound) and baseline. The neural oscillatory activities from repetitive movement events were overlaid with each other and were observed as cyclic amplitude changes which usually returned to their baselines (see Fig. 2). Therefore, we adopt the method slightly modified from Toma *et al*.^18^. The oscillatory responses of repetitive movements were quantified by calculating the amplitude ratio (*PAmp*_*resp*_) between amplitude difference (*Amp*_*max*_ − *Amp*_*min*_) and the mean of event-related oscillatory response, *i.e*., (*Amp*_*max*_ + *Amp*_*min*_)/2. The *PAmp*_*resp*_s were then used to quantify and compare among oscillatory responses induced by different movement rates in healthy subjects and stroke patients.

Movement rate has been regarded as a critical factor to affect brain oscillatory activities^1^,^2^,^3^,^18^,^19^,^20^,^28^,^29^,^30^,^31^,^32^. Toma *et al*.^18^ studied the cortical network changes in slow-rate and fast-rate movements. They suggested the observation of larger oscillatory activity change in slow-rate movement (IMI ≥ 1 s) is due to the requisite deactivation and decoupling of cortical idling in slow-rate movement, in order to separate individual movement. In fast-rate movement (IMI < 1 s), several cortical areas, such as frontal area, supplementay motor area, primary motor area, angular gyrus, etc.^3^,^20^, are strongly coupled and activated which results in less cognitive demand and fewer neuronal ensembles^19^. Our observations are consistent with previous literatures (see Fig. 3) which reported both the alpha and beta *PAmp*_*resp*_s were decreased with the increase of movement rate^18^,^19^,^29^,^32^. Since the alpha-band of sensorimotor Mu rhythm is more associated with motor preparation and its beta-band portion is relevant to motor idling or inhibition, the greater suppression in alpha *PAmp*_*resp*_ for fast movement might reflect the transition from DMN to automatic timing which resulted in the reduced cognitive demands for motor planning in fast-rate movements^4^. Besides, it can be observed that the *PAmp*_*resp*_s were contralateral dominant in both alpha and beta bands which could indicate the timing control of repetitive movement is mainly initialized on the hemisphere contralateral to movement hand.

It was of interest to see that discrepancy was found between the results of non-paretic and paretic hand movements in stroke patients. In the results of paretic hand movements (see Figs 4b and 5b), the alpha and beta *PAmp*_*resp*_s showed no difference among different movement rates. Compared to the movements of non-paretic hand (see Fig. 4a,b), the alpha and beta *PAmp*_*resp*_s were similar to the observations in healthy subjects (Fig. 3) in which the alpha and beta *PAmp*_*resp*_s in slow-rate and moderate-rate movements were significantly larger than those in fast-rate movements. It might imply the stroke patients require more cognitive demands than healthy subjects in performing the fast-rate movement task. According to Poppel’s study^4^, the neural network is shifted from DMN to automatic timing with the increase of movement rate. The higher *PAmp*_*resp*_s in stroke patients could be due to the impairment of neural network for automatic timing which might break down the repetitive movements into discrete movements.

It has been reported in many studies that enhanced excitability can be observed in unaffected hemisphere which induces the inter-hemispheric inhibition driven from the unaffected to the affected hemisphere^26^. The hyperactivities in unaffected hemisphere result in the laterality dominance of alpha ERD^17^. This is coherent with our observations that the alpha and beta *PAmp*_*resp*_s were ipsilaterally dominant in paretic hand movements (see Figs 4b and 5b). According to the previous literatures, the alpha and beta bands in sensorimotor Mu rhythm can express distinct functions in achieving a motor execution, the alpha-band sensorimotor Mu rhythm is more related to motor preparation and the beta-band sensorimotor Mu rhythm is correlated with proprioceptive afferent input^33^. The dominance of unaffected hemisphere in alpha band might be caused by the increase of cognitive efforts to move paretic hand, and the ipsilateral dominance of beta *PAmp*_*resp*_s in paretic hand movement could reflect the loss of proprioceptive afferent input from affected limbs^33^.

The *LI*s of alpha and beta *PAmp*_*resp*_s shown in Fig. 6 demonstrate the dominance of unaffected hemisphere in stroke patients. Unlike the *LI*s in hand movements of healthy subjects and the non-paretic hands in stroke patients, the *LI*s of paretic hand movements in stroke patients showed laterality dominance in ipsilateral hemisphere both in alpha and beta bands which is in line with the observation in other literatures^24^,^34^. In addition, it might be worthy to notice that the laterality of beta *PAmp*_*resp*_s was inclined more contralaterally in the hand movements of healthy subject and the non-paretic hands in stroke patients. The movement-rate dependent laterality echoes the fMRI study proposed by Agnew *et al*.^35^ who observed the percentage of signal changes were enhanced with the increase of movement rate in posterior putman and contralateral thalamus. The enhanced activities in subcortical structures could be activated by both deep joint receptors and cutaneous receptors during finger movements. Since beta oscillatory activity in sensorimotor Mu rhythm is related to proprioceptive input, the laterality change of beta *PAmp*_*resp*_ in our study might reflect the increase of peripheral afferent input during fast repetitive movements.

**Figure 7:**
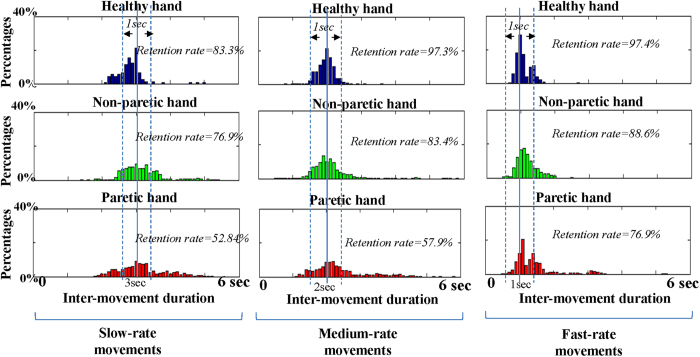
The histograms of percentages of inter-movement durations executed by healthy and stroke subjects.

This study examined the responses of event-related oscillatory activities induced by different movement rates in stroke patients. We considered the execution of repetitive movements in stroke patents was not as stable as healthy subjects. The inter-movement duration was calculated as an index to pick up suitable epochs (IMI with deviation time < 0.5 s) for *PAmp*_resp_ calculation. It can be observed in Fig. 7 that the retention rate was negatively proportional to the inter-movement duration which reflected the difficulty in reproducing the volitional control of long IMI movements. It echoed the Poppel’s study that the capability of time estimation in human was tested by providing a stimulus reproduction task from 0.5 s to 7 s. They found movements became temporally irregular for IMI above 3 s and indicated precise control of movement with IMI longer than 3 s is not possible^4^.

## Conclusions

This paper studied the oscillatory activities induced by different movement rates in healthy subjects and chronic stroke patients. Percentage changes of amplitude response *PAmp*_*resp*_s in the hand movements of healthy subjects and the non-paretic and paretic hand movements in stroke patients were compared. In our study, several findings in alpha and beta *PAmp*_*resp*_s were found. First, the alpha and beta *PAmp*_*resp*_s declined with the increase of movement rates in healthy subjects. Second, the *PAmp*_*resp*_s in slow-rate and moderate-rate movements were significantly larger than those in fast-rate movements when stroke patients were moving their non-paretic hands, whereas no difference was found among the *PAmp*_*resp*_s obtained from the three movement rates in paretic hand movements. Third, the *PAmp*_*resp*_s were contraleral dominance in the hand movements of healthy subjects and the non-paretic hand movements in stroke patients. However, ipsilateral (unaffected side) dominance in alpha and beta *PAmp*_*resp*_ was found when stroke patients were moving their paretic hands. Since different neural network systems might be engaged in the transition from slow-movement to fast- movement rates, the high *PAmp*_*resp*_s in paretic hand movements might indicate the incapability of involving automatic timing mechanism or the requirement of involving extra cognitive efforts to maintain the fast-rate movements. Our study has demonstrated that repetitive movement could be a choice to probe the function of abnormality in lesional hemisphere. Further investigation is needed to discover the discrepancy in mechanisms of neural networks between healthy subjects and stroke patients.

## Additional Information

**How to cite this article**: Hsu, C.-C. *et al*. Study of Repetitive Movements Induced Oscillatory Activities in Healthy Subjects and Chronic Stroke Patients. *Sci. Rep.* **6**, 39046; doi: 10.1038/srep39046 (2016).

**Publisher's note:** Springer Nature remains neutral with regard to jurisdictional claims in published maps and institutional affiliations.

## Figures and Tables

**Table 1:** Demographic Data Of Stroke Patients.

#	Age	Sex	Months after stroke	Grip force (kg)		Main lesion site identified on CT scan	Affected Hemisphere	Affected hand
				Paretic hand	Non-paretic hand			
P1	46	M	10	2.3	17	BG, Th	R	L
P2	47	F	8	1.7	5.6	PC	L	R
P3	62	M	6	15.3	16.7	BG	L	R
P4	61	M	6	3.1	18	PC	L	R
P5	54	M	72	3.5	33.3	PC	L	R
P6	63	M	28	6.7	11	BG	L	R
P7	63	M	36	8.8	28.8	PC	R	L
P8	52	M	58	2.4	30.7	PC	R	L
P9	63	F	6	1.1	10.2	BG	R	L
P10	64	M	6	0.9	5.6	BG	L	R
P11	47	M	8	1.3	13.2	Th	L	R
P12	61	M	10	1.9	19	BG	R	L
P13	62	M	15	2.2	22	FC	R	L
P14	53	M	7	0.9	9.7	PC	L	R
P15	59	M	8	1.5	29	PC	L	R
P16	57	M	6	0.7	22.4	BG	L	R
P17	54	F	6	0.9	11	BG	R	L
P18	64	F	16	3.3	13.3	BG	R	L
P19	61	F	9	1,1	17.1	TH	R	L
P20	72	M	31	5.2	21.2	PC	L	R
Mean ± Std.	58.3 ± 6.8		17.6 ± 18.7	3.4 ± 3.6	17.7 ± 8.1			

*R* = *right; L* = *left; BG* = *basal ganglia; FC* = *frontal cortex; PC* = *parietal cortex; Th* = *thalamus.*
